# The impact of clinical seizures and adverse brain MRI patterns in neonates with hypoxic-ischemic encephalopathy and abnormal neurodevelopment

**DOI:** 10.1016/j.clinsp.2024.100533

**Published:** 2025-01-02

**Authors:** Sae Yun Kim, Hyun-Mi Kang, Soo-Ah Im, Young-Ah Youn

**Affiliations:** a Department of Pediatrics, Seoul St. Mary's Hospital, College of Medicine, The Catholic University of Korea, Seoul, South Korea; b Department of Radiology, Seoul St. Mary's Hospital, College of Medicine, The Catholic University of Korea, Seoul, South Korea

**Keywords:** Hypoxia-ischemia, Therapeutic hypothermia, Neurodevelopmental impairment, Seizure, Diffusion weighted MRI

## Abstract

**Introduction:**

This study aimed to investigate the associations among seizures, clinical characteristics, and brain injury on Magnetic Resonance Imaging (MRI) in infants with Hypoxic Ischemic Encephalopathy (HIE), and to determine whether these findings can predict unfavorable neurodevelopmental outcomes.

**Method:**

Clinical and electrographic seizures were assessed by amplitude-integrated electroencephalogram, and the extent of brain injury was evaluated by using MRI. At 12‒24 months of age, developmental impairment or death was assessed. Between 2012 and 2020, 143 newborns were admitted for HIE, and 8 infants were excluded from the study.

**Results:**

Eighty-five infants were diagnosed with greater than moderate HIE and 65 infants underwent therapeutic hypothermia. In addition, 38 infants experienced clinical seizures (clinical seizure group, CSG), 49 infants had electrographic seizures (Electrographic Seizure Group, ESG), and 48 infants had no seizures (no seizure group, NSG). The proportion of infants with neurodevelopmental impairment or death was significantly higher in the CSG than in the NSG (57.7 % and 26.1 %, *p* = 0.026). A risk factor analysis indicated that cord blood pH (adjusted Odds Ratio [aOR = 0.01]; 95 % Confidence Interval [95 % CI 0.001‒0.38]; *p* = 0.015) and MRI findings (aOR = 4.37; 95 % CI 1.25‒15.30; *p* = 0.012) were independently associated with abnormal neurodevelopment, after adjustment.

**Discussion:**

Clinical seizures in infants with HIE were independently associated with abnormal neurodevelopment. However, cord blood pH and abnormal brain MRI findings were consistently linked to long-term neurodevelopmental outcomes.

## Highlights

• Electrographic seizures did not have predictive value for adverse neurodevelopmental outcomes at 12‒24 months of age.
• The incidence of neurodevelopmental impairment or death was significantly higher in the clinical seizure group.
• Only cord blood pH and abnormal brain MRI findings were consistently associated with long-term neurodevelopmental outcomes in a multivariate analysis.

## Introduction

Seizures are the most common manifestations of neurologic disease in the neonatal period, occurring in approximately 3.5 newborns per 1000 live term births.^1^ The etiology of neonatal seizures is most commonly associated with Hypoxic-Ischemic Encephalopathy (HIE), accounting for >60 % of early onset seizures.^1^^,^^2^ HIE is a brain injury that prevents adequate blood flow to an infant's brain as a result of a hypoxic-ischemic event that occurs during the prenatal, intrapartum, or postnatal period.^3^ HIE after perinatal asphyxia affects 1.0 to 8.0 cases per 1000 live births^4^ and is an important cause of morbidity and mortality in newborns.^5^^,^^6^ From a recent large cohort study in Brazil, seizures were identified in 33.9 % of the infants with HIE.^7^

High seizure burden has been associated with worse neurologic outcomes and subsequent epilepsy.^8^ Hence, seizures resulting from HIE may serve as indicators of the severity of underlying brain injuries and are known to be associated with adverse neurological development. Because of the nature of subtle neonatal seizures, their detection can be challenging for clinicians. For example, subclinical seizures, manifest solely on Electroencephalogram (EEG) recordings. Furthermore, clinical detection is often more difficult due to the intermittent and transient nature of seizures in neonates.^9^^,^^10^ Previously, Murray et al. reported that most neonatal seizures are electrographic-only events without clinical symptoms.^11^ However, if seizures are diagnosed by EEG only, sudden alterations in heart rate or blood pressure may be attributed to seizures in Neonatal Intensive Care Unit (NICU) settings, although these alterations could also arise from normal physiological responses such as paroxysmal autonomic movements. Accordingly, there is a particularly high risk of overdiagnosis in critically ill neonates, with some benign movements erroneously labeled as seizures.^12^^,^^13^ On the other hand, relying solely on clinical signs may result in many seizures being missed. For these reasons, bedside neuromonitoring with Amplitude-integrated EEG (aEEG) has been widely adopted in NICUs to achieve recognition of most electrographic seizures. The accuracy of neonatal seizure diagnosis can be improved by monitoring both clinical and electrographic seizures.

Magnetic Resonance Imaging (MRI) is very useful for predicting the neurodevelopmental outcomes of infants with HIE. MRI findings are frequently utilized as a potential prognostic indicators for the neurodevelopmental outcomes of neonates with moderate-to-severe HIE.^14^ The pattern of brain injury is related to the duration and severity of the hypoxic-ischemic event. The quantification of the extent of brain injury via a standardized MRI scoring system is highly important for the prediction of long-term neurodevelopmental outcomes in neonates with moderate and severe HIE. The present study aimed to investigate the clinical characteristics, and neonatal outcomes including mortality, of infants in different seizure groups. The secondary goal was to determine the predictive value of the clinical features for adverse neurodevelopmental outcomes at 12‒24 months of age.

## Materials and methods

### Study design and patient enrollment

This was a retrospective cohort study of infants who were diagnosed with HIE. The authors included term or late preterm infants (≥ 35 weeks of gestation with a birth weight of ≥ 1800 g) with HIE who were born between April 2012 and October 2020 at Seoul St. Mary's Hospital, which is a tertiary referral university hospital with a level IV NICU. The evidence of moderate or severe encephalopathy was distinguished using Sarnat clinical stages.^15^ All infants who experienced acute perinatal events were recruited and assessed for signs of HIE. Enrolled infants had to meet one of the following: (1) An Apgar score of 5 at 10 min after birth, (2) The continued need for resuscitation at 10 min after birth, or (3) A pH ≤ 7.0 or a base deficit > 16 mmoL/L on an umbilical cord blood sample or an arterial or venous blood sample obtained within 60 min of birth. The authors excluded infants with HIE who had major congenital abnormalities, syndromes, or metabolic diseases. Infants with a birth weight ≤ 1800 g, a Gestational Age (GA) < 35 weeks, overt bleeding, and signs of infection were also excluded. When infants met greater than moderate HIE criteria, they were subjected to Therapeutic Hypothermia (TH) within 6 h of birth: whole-body cooling (Blanketrol III Hyper-Hypothermia System, Cincinnati Sub-Zero, Cincinnati, OH) with the core esophageal temperature maintained at 33.5 °C for 72h

### Definitions of clinical seizures and electrographic seizures

Seizures were clinically diagnosed by an attending neonatologists or pediatric neurologists as a paroxysmal alterations in motor function; these alterations included clonic, tonic, and subtle seizure manifestations.^16^ All included infants were assessed using Cerebral Function Monitoring (CFM) (CFM, Natus Medical Inc., Seattle, WA) or video EEG as early as possible.^17^ EEG recordings continued for at least the first several hours or until a neonatologist or pediatric neurologist decided that no more electrographic seizures were being detected. Moderate or severe voltage changes on aEEG or electrographic seizure waves were considered electrographic seizures.^18^

Infants with clinical seizures and infants with both clinical and electrographic seizures were included in the Clinical Seizure Group (CSG). The authors regarded mortality as the most severe outcome of clinical seizures, and infants who died due to HIE were also included in the CSG. For infants with available CFM or video EEG data, only those with electrographic seizures were detected without clinical seizures were included in the ESG. Finally, infants with neither electrographic nor clinical seizures were included in the NSG.

### MRI acquisition and analyses

**Fig. 1:**
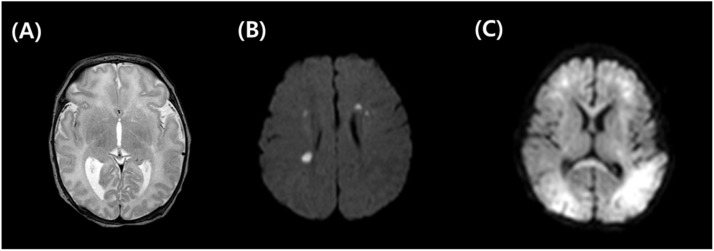
**Comparison of normal vs. abnormal brain MRI.** Normal brain MRI, T2 weighted (A), abnormal brain MRI with multifocal periventricular white matter lesions, diffusion weighted image (B), abnormal brain MRI with global ischemic change both subcortical and white matter, diffusion weighted image (C).

If infants were diagnosed with moderate to severe HIE, brain MRI with diffusion was performed as soon as the infants no longer required intubation. MR images were reviewed and categorized by a pediatric radiologist with over 20 years of work experience, who was blinded to the treatment and outcomes of the infants. The MRI National Institute of Child Health and Human Development (NICHD) scoring system was used for HIE evaluation. The authors classified the MRI findings according to the NICHD pattern for brain injury: a score of 0 was given for a normal MR image; a score of 1A was given for only minimal cerebral lesions; a score of 1B was given for more extensive cerebral lesions without the involvement of the Basal Ganglia and Thalamus (BGT), or the Posterior Limb of the Internal Capsule (PLIC), or the Anterior Limb of Internal Capsule (ALIC) involvement and no area of watershed infarction; a score of 2A was given for any BGT, PLIC, or ALIC involvement or watershed infarction but no cerebral lesions; a score of 2B was given for any BGT, PLIC, or ALIC involvement or watershed infarction with additional cerebral lesions; and a score of 3 was given for cerebral hemispheric devastation.^19^ Fig. 1 shows the comparison between normal and abnormal brain images with multifocal white matter lesions or global ischemic lesions.

### Long-term neurodevelopmental outcomes

Surviving infants returned for follow-up evaluations, the developmental assessments were completed when the infants were aged at 12–24 months. Developmental delay was defined when at least one of the following criteria was met: (1) The Korean version of the Bayley Scales of Infant and Toddler Development-Third Edition Cognitive Composite score, Language Composite score, or Motor Composite score of < 85^20^ (2) The Korean Developmental Screening Test for Infants and Children score was below −2 standard deviations in one or more domains^21^ and (3) The scores of the Korean Ages & Stages Questionnaire dimensions fell below the cut-off scores in one or more areas.^22^ Developmental delay or epilepsy/cerebral palsy was considered neurodevelopmental impairment. The primary outcome of this study was abnormal Neurodevelopment (ND) which was a composite of long-term neurodevelopmental impairment and/or death.

### Statistical analysis

Data are expressed as numbers with percentiles (%) or medians with interquartile ranges. Categorical variables were analyzed using the χ^2^ test or Fisher's exact test, as appropriate, and continuous variables were analyzed using Mann-Whiteny test, because they were not normally distributed but skewed. The authors compared the baseline characteristics between infants in the CSG and infants in the NSG, and between ESG and NSG. The authors compared the baseline characteristics of infants in the CSG and those of infants in the NSG. And the analysis compares the baseline characteristics of infants in the ESG and those of infants in the NSG, separately. For multivariate logistic regression analyses, covariates were selected among the factors that showed independent differences, with a *p* < 0.05, between the groups with normal ND group and abnormal ND. Standard regression analysis was conducted, by the “Enter” method in SPSS. All statistical analyses were two-tailed, with statistical significance defined as a value of *p* < 0.05. All statistical analyses were performed with SPSS version 25 (IBM Corp, Armonk, New York, USA).

## Results

Between April 2012 and October 2020, a total of 143 newborns with acute perinatal events were admitted for HIE, and after exclusion, 135 infants were analyzed. Among the 135 infants, 85 (63.0 %) infants were diagnosed with greater than moderate HIE; however, because of severe clinical conditions, TH could not be performed to 15 infants with moderate HIE and 22 infants with severe HIE.

**Fig. 2:**
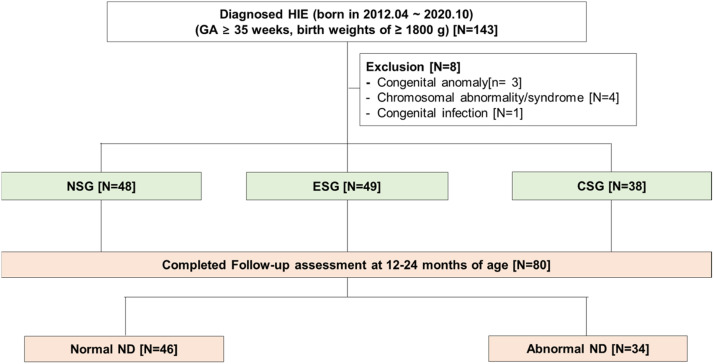
**Flow chart of the study population from birth to follow-up.** CSG, Clinical Seizure Group; ESG, Electrographic Seizure Group; HIE, Hypoxic Ischemic Encephalopathy; ND, Normal Development; NSG, No Seizure Group.

Thirteen infants were diagnosed with severe HIE, and 1 infant with moderate HIE did not undergo an MRI scan before discharge because of their clinical condition. Additionally, twenty-six infants with mild HIE were discharged without an MRI scan. Forty-eight infants were discharged without seizure, and electrographic seizures were detected in 49 infants. Thirty infants experienced definite clinical seizures and 8 infants died due to HIE, finally, 38 infants were included in the CSG. A total of 80 infants completed the follow-up assessment at 12–24 months of age including those who died (Fig. 2).

### Comparison of the characteristics according to seizure group

The descriptive clinical characteristics of infants with HIE in the CSG and ESG are presented in Table 1. There were no significant differences between the seizure groups and the NSG in terms of maternal intrapartum complications, except for fetal decelerations, and emergency cesarean section. Fetal deceleration was detected more often in the mothers of infants with seizures than in the mothers of infants without seizures: 18.8 % (9/48), in the NSG, 38.8 % (19/49) in the ESG, and 60.5 % (23/38) in the CSG (*p* = 0.03, and *p* < 0.001 for comparison with the ESG, and the CSG respectively). The CSG had the highest proportion of infants who were delivered by emergency Cesarean section, followed by the ESG, and the NSG (60.5 %, 30.6 %, and 18.8 % respectively). However, the differences were significant only between the CSG and the NSG (*p* < 0.001).

**Table 1:** Characteristics of study population: comparison according to seizure.

	NSG [*n* = 48]	ESG [*n* = 49]	ap	CSG [*n* = 38]	^b^p
**Maternal characteristics**					
Maternal age	33.5 [29, 41]	34 [21, 40]	0.720	34 [24, 39]	0.879
Fetal decelerations	9 (18.8 %)	19 (38.8 %)	0.030	23 (60.5 %)	<0.001
Uterine rupture	0 (0.0 %)	0 (0.0 %)	‒	0 (0.0 %)	‒
Maternal pyrexia	0 (0.0 %)	0 (0.0 %)	‒	2 (5.3 %)	0.192
Maternal hemorrhage	2 (4.2 %)	5 (10.25)	0.436	3 (7.9 %)	0.651
Premature rupture of membrane	8 (16.7 %)	4 (8.2 %)	0.203	5 (13.2 %)	0.652
Emergency cesarean section	9 (18.8 %)	15 (30.6 %)	0.176	23 (60.5 %)	<0.001
**Neonatal characteristics**					
Gestational age, weeks	384/7 [350/7, 412/7]	393/7 [351/7, 413/7]	0.354	400/7 [352/7, 411/7]	0.397
Birth weight, g	3330 [2020, 3960]	3100 [2240, 4060]	0.201	3200 [1880, 4120]	0.182
Small for gestational age	2 (4.2 %)	3 (6.1 %)	>0.999	6 (15.8 %)	0.131
Inborn	21 (43.8 %)	22 (44.9 %)	>0.999	14 (36.8 %)	0.517
Male sex	32 (66.7 %)	26 (53.1 %)	0.172	22 (57.9 %)	0.403
5 min Apgar Score	9 [2, 10]	8 [1, 10]	0.029	7 [0, 10]	<0.001
10 min Apgar Score < 7	8 (16.7 %)	12 (24.5 %)	0.341	17 (44.7 %)	0.004
Cord blood pH	7.30 [7.09, 7.51]	7.33 [6.91, 7.55]	0.877	7.28 [6.53 7.61]	0.210
Sarnat stage, 1st day ≥ moderate	13 (27.1 %)	36 (73.5 %)	<0.001	36 (94.7 %)	<0.001
Therapeutic hypothermia	7 (14.6 %)	30 (61.2 %)	<0.001	25 (65.8 %)	<0.001
Abnormal background pattern on aEEG	3 (6.3 %)	25 (51.0 %)	<0.001	18 (47.4 %)	<0.001
AED use	5 (10.4 %)	47 (95.9 %)	<0.001	34 (89.5 %)	<0.001

Values are presented as numbers (percentages) for categorical variables and medians (Interquartile Ranges [IQRs]) for continuous variables, as appropriate.

a p-values for comparing the independent associations of the ESG and NSG, and ^b^ p-values for comparing the independent association of the CSG and NSG, which were calculated by using Chi-Square test or Fisher's exact test for categorical variables, and Mann-Whiteney *U* test for continuous variable, as appropriate. AED, Antiepileptic Drug; CSG, Clinical Seizure Group; ESG, Electrogrphic Seizure Group; NSG, No Seizure Group; TH, Therapeutic Hypothermia; aEEG, Amplitude Electroencephalograpy.

The median GA at birth and birth weight were not significantly different among the groups. Furthermore, the median value of 5 min Apgar score was significantly lower in both seizure groups than in the NSG: 9^,^^2^ ^10^ for the NSG, 8^,^^1^ ^10^ for the ESG, and 7 [0, 10] for the CSG (*p* = 0.029, and *p* < 0.001 for comparison with the ESG and the CSG, respectively). The CSG had the highest proportion of infants whose Apgar score was < 7 at 10 min, followed by the ESG and the NSG (44.7 %, 24.5 %, and 16.7 % respectively). However, the differences were significant only between the CSG and the NSG (*p* = 0.004). According to the initial neurological exam, the proportion of infants with moderate to severe HIE according to Sarnat stage on the first day was highest in the CSG, followed by the ESG, and the NSG (94.7 %, 73.5 %, and 27.1 % respectively), and both differences were significant (both *p* < 0.001). Consequently, 64 (47.4 %) infants underwent TH: 7 in the NSG, 30 in the ESG, and 25 in the CSG. Electrographically, the proportion of infants with abnormal background patterns on aEEG was highest in the ESG (51.0 %), followed by CSG (47.4 %), and the NSG (6.3 %).

**Fig. 3:**
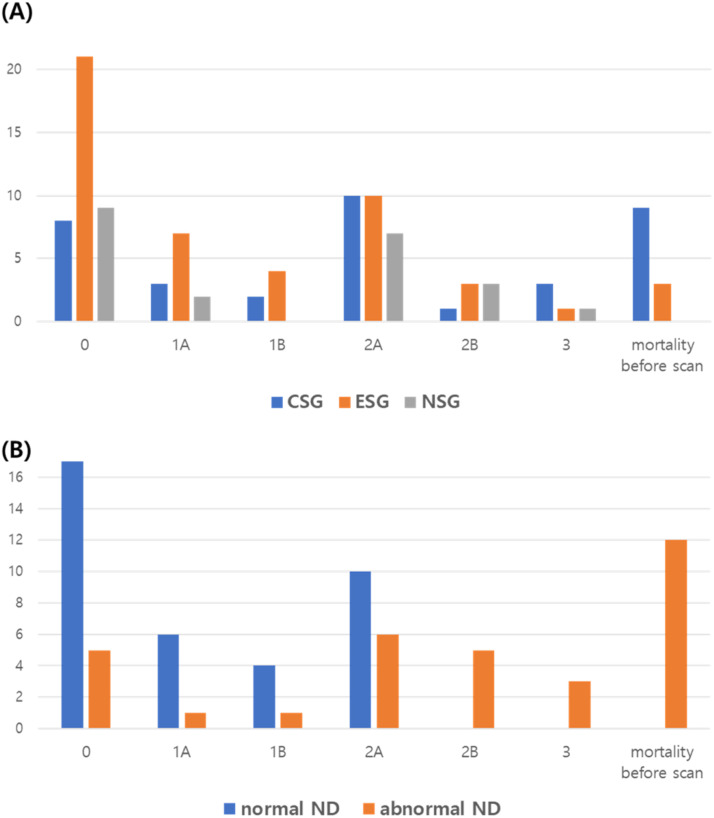
**NICHD MRI score.** Among the 80 infants who were able to be assessed at 12–24-months of age, all infants in the normal neurodevelopment group discharged after MRI scan. However, 12 infants discharged without MRI scans. NICHD MRI grade were visualized according to seizure group (A), and developmental group (B). Two infants in the clinical seizure group were discharged without MRI scans, and 26 infants in the no-seizure group did not undergo MRI scans. CSG, infant with Clinical Seizure Group; ESG, infants with Electrographic Seizure Group; ND, Neurodevelopment; NSG, Infant without Seizure Group.

The percentage of infants who developed hypotension requiring inotropic agent therapy was the highest in the CSG (68.4 %), followed by the ESG (63.3 %), and the NSG (33.3 %) (*p* = 0.003 between the NSG and the ESG, *p* = 0.001 between the NSG and the CSG). When the NICHD MRI score of 0 considered a normal MRI finding and others were considered an abnormal MRI finding, the percentage of infants with abnormal MRI findings did not differ according to seizure group. The length of hospital stay was not different among the three groups. All causes of mortality were significantly higher in the CSG (26.3 %) than in the NSG (0.01 %) (*p* < 0.001). Primary outcomes were analyzed among survivors, and both neurodevelopmental impairment and the composite outcome with death were significantly higher in the CSG than in the NSG (57.7 %, and 26.1 %, respectively, *p* = 0.026). Furthermore, there were not a significant difference between the ESG and the NSG (Table 2 and Fig. 3).

**Table 2:** Neonatal outcome: comparison according to seizure.

	NSG [*n* = 48]	ESG [*n* = 49]	ap	CSG [*n* = 38]	^b^p
PPHN	7 (14.6 %)	15 (30.6 %)	0.059	10 (26.3 %)	0.175
Hypotension	16 (33.3 %)	31 (63.3 %)	0.003	26 (68.4 %)	0.001
Abnormal MRI	13 (59.1 %)	28 (57.1 %)	0.878	28 (77.8 %)	0.129
Length of stay, days	10 [4, 58]	14 [4, 56]	0.719	12 [1, 146]	0.230
Mortality before discharge	0 (0.0 %)	3 (6.1 %)	0.242	10 (26.3 %)	<0.001
**FU assessment at 12‒24 months of age**	[*n* = 23]	[*n* = 31]		[*n* = 26]	
Neurodevelopmental impairment	6/23 (26.1 %)	12/31 (38.7 %)	0.289	15/26 (57.7 %)	0.026
Neurodevelopmental impairment or death at 12‒24m	6/23 (26.1 %)	13/31 (41.9 %)	0.228	15/26 (57.7 %)	0.026

Values are presented as numbers (percentages) for categorical variables and medians (Interquartile Ranges [IQRs]) for continuous variables, as appropriate.

a p-values for comparing the independent associations of the ESG and NSG, and ^b^p-values for comparing the independent association of the CSG and NSG, which were calculated by using Chi-Square test or Fisher's exact test for categorical variables, and Mann-Whiteney *U* test for continuous variable, as appropriate. CSG, Clinical Seizure Group; ESG, Electrogrphic Seizure Group; MRI, Magnetic Resonance Imaging; NSG, No Seizure Group; PPHN, Persistent Pulmonary Hypertension of the Newborn.

### Risk factors for abnormal ND at 12–24 months of age according to a multivariable logistic regression analysis

Among 80 infants who completed the long-term assessment 12–24 months of age, 46 infants did not show developmental delay, epilepsy, or cerebral palsy, and they were included in the normal ND group. And 34 infants were defined as having neurodevelopmental impairment or death, therefore they were classified as abnormal ND group. According to the univariate analyses, a greater proportion of mothers of infants in the abnormal ND group (18/34, 52.9 %) than that of the normal ND group (14/46, 30.4 %) underwent emergency Cesarean section (*p* = 0.042). The median value of cord blood pH was 7.30 (6.95, 7.50) for the normal ND group and 7.21 (6.53, 7.39) for the abnormal ND group (*p* = 0.001). A greater percentage of infants in the abnormal ND group than in the normal ND group were assessed as having *a* ≥ moderate Sarnat stage on the first day of life (29/34 [85.3 %], and 30/46 [65.2 %] respectively, *p* = 0.044). Persistent pulmonary hypertension of the newborn also occurred more frequently in the abnormal ND group than in the normal ND group (13/34 [38.2 %] and 8/46 [17.4 %], respectively; *p* = 0.036). Abnormal MRI findings according to the NICHD score were analyzed among infants who had MRI scan data; 20/37 (54.1 %) infants in the normal ND group, and 28/33 (84.8 %) infants in the abnormal ND group had abnormal MRI (*p* = 0.006). These five factors that showed significant differences in the univariate analyses were selected as potential confounders. Through multivariate logistic regression analyses, the authors further investigated the impact of potential confounding factors on abnormal ND. Infants with a higher cord blood pH had a lower risk of abnormal ND (adjusted Odds Ratio [aOR = 0.01]; 95 % Confidence Interval [95 % CI 0.001–0.38]; *p* = 0.015). Infants with abnormal MRI findings had 4.37 fold greater risk of abnormal ND (aOR = 4.37; 95 % CI 1.25–15.30; *p* = 0.012) (Table 3).

**Table 3:** Comparison of normal/abnormal neurodevelopmental groups.

	Normal ND [*n* = 46]	Abnormal ND [*n* = 34]	OR (95 % CI)	p	aOR (95 % CI)	ap
Maternal characteristics						
Maternal age, years	33.0 [26,41]	36 [24, 40]		0.054		
Fetal decelerations	17 (37.0 %)	13 (38.2 %)	1.06 (0.42, 2.64)	0.907		
Uterine rupture	0 (0.0 %)	0 (0.0 %)	‒	‒		
Maternal pyrexia	1 (2.2 %)	0 (0.0 %)	‒	>0.999		
Maternal hemorrhage	3 (6.5 %)	5 (14.7 %)	2.47 (0.55. 11.52)	0.275		
Premature membrane rupture	4 (8.7 %)	4 (11.8 %)	1.40 (0.32, 6.05)	0.717		
Emergency Cesarean section	14 (30.4 %)	18 (52.9 %)	2.57 (1.02, 6.46)	0.042	1.16 (0.37, 3.69)	0.797
**Neonatal Characteristics**						
Gestational age, weeks	39^3/7^ [35°^/7^, 41^3/7^]	38^6/7^ [35^1/7^, 41°^/7^]	‒	0.362		
Birth weight	3110 [2448, 4060]	3285 [2240, 4120]	‒	0.540		
Small for gestational age	4 (8.7 %)	1 (2.9 %)	3.14 (0.34, 29.47)	0.388		
Inborn	21 (45.7 %)	23 (67.6 %)	2.49 (0.99, 6.27)	0.051		
Male sex	21 (45.7 %)	13 (38.2 %)	0.74 (0.30, 1.82)	0.507		
5-minutes Apgar Score	8[2, 10]	7 [0, 10]	‒	0.082		
10-minutes Apgar Score <7	15 (32.6 %)	12 (35.3 %)	1.13 (0.44, 2.87)	0.802		
cord blood pH	7.30 [6.95, 7.50]	7.21 [6.53, 7.39]	‒	0.001	0.01 (0.001, 0.38)	0.015
Sarnat stage, 1st day ≥ moderate	30 (65.2 %)	29 (85.3 %)	3.09 (1.00, 9.54)	0.044	1.01 (0.22, 4.60)	0.990
Therapeutic hypothermia	24 (52.2 %)	18 (52.9 %)	1.03 (0.42, 2.51)	0.946		
Clinical seizure	27 (58.7 %)	17 (50.0 %)	1.42 (0.58, 3.47)	0.440		
Electrographic seizure on aEEG	24 (52.2 %)	22 (64.7 %)	1.68 (0.68, 4.18)	0.262		
Hypotension	27 (58.7 %)	22 (27.5 %)	1.29 (0.52, 3.23)	0.585		
AED use	31 (67.4 %)	25 (73.5 %)	1.34 (0.50, 3.58)	0.554		
PPHN	8 (17.4 %)	13 (38.2 %)	2.94 (1.05, 8.23)	0.036	1.97 (0.57, 6.80)	0.286
Length of stay	12 [4,35]	13.5[1, 146]	‒	0.664		
Abnormal MRI findings	20/37 (54.1 %)	28/33 (84.8 %)	4.76 (1.51, 15.04)	0.006	4.37 (1.25, 15.30)	0.012

Values are presented as numbers (percentages) for categorical variables and medians (Interquartile Ranges [IQRs]) for continuous variables, as appropriate. p-values were calculated by using Chi-Square test or Fisher's exact test for categorical variables, and Mann-Whiteney *U* test for continuous variable, as appropriate.

a p-values were calculated by a multivariate logistic regression model, adjusted for multiple confounders: emergency Cesarean section, 10-minutes Apgar Score <7, Sarnat stage, 1st day ≥ moderate, PPHN, and abnormal MRI. AED, Antiepileptic Drug; aEEG, Amplitude Electroencephalograpy; aOR, Adjusted Odd Ratios; CI, Confidence Interval; MRI, Magnetic Resonance Image; PPHN, Persistent Pulmonary Hypertension of the Newborn.

## Discussion

This study provides information for assessing both clinical and electrographic seizures in asphyxiated neonates and significantly contributes to the literature by emphasizing the importance of seizure patterns and MRI findings. The present study has several notable findings. First, more infants in the CSG than in the NSG experienced neurodevelopmental impairment or death. However, there were no significant differences in long-term neurodevelopmental outcomes at 12–24 months of age between the NSG and ESG. Second, the initial cord blood pH and MRI findings were independently associated with long-term neurodevelopment in asphyxiated infants.

It is crucial to understand seizures in newborn infants because they are frequently followed by various unfavorable outcomes.^23^ From a recent systematic review, the presence of neonatal seizures in term newborns with vascular or hypoxic brain injury may have an impact on or be a predictor of neurodevelopmental outcomes.^24^

Many neonatal seizures are subclinical; thus, bedside neuromonitoring with aEEG has been widely adopted in NICU settings.^25^ Clinical or electrographic seizures have different long-term effects on neonates. In a previous small retrospective study in Turkey, Kurul and colleagues reported that psychomotor retardation or epilepsy at 1 year of age was not observed in infants who had electrographic seizures without clinical seizures. In Contrast, 53.8 % of infants who have both electrographic and clinical seizures developed psychomotor retardation at 1 year of age, and one infant developed epilepsy.^26^ Recently, Hunt et al. conducted a large randomized controlled trial and compared the odds of death or disability at 2 years of age between infants treated for both clinical and electrographic seizures and infants treated for clinical seizure only, and they reported that there were no significant differences between the groups.^27^ A systematic review did not identify evidence demonstrating improved neurodevelopmental outcomes or improved survival in neonates treated for electrographic seizures versus those treated for clinical seizures.^28^ These findings suggest that, regardless of whether electrographic seizures detected by aEEG are treated, there is no difference in long-term neurodevelopment. This study also demonstrated that more infants in the CSG than in the NSG exhibited neurodevelopmental impairment or death. In other words, electrographic seizures have little effect on long-term neurodevelopment, which is partially consistent with the present result.

Because electrographic seizures do not significantly affect long-term neurodevelopment, it is still not known whether earlier treatment of electrographic seizures will alter the course of the outcomes and result in less severe brain injury. Compared with the developed brain, the ability of the immature brain to generate seizures is linked to a series of unique developmental features with increased excitability and to a greater risk of sustaining brain injury in newborns. For instance, Glass et al.^29^ reported that recurrent seizures in infants with HIE may increase injury to the developing brain. Accordingly, seizures in neonates can impede normal development of the brain and may reduce the efficiency of cortical networks, even in the absence of cell loss.^30^^,^^31^ For this reason, Young et al. in a 2016 Cochrane's review^32^ reported a reduced risk of seizures in 147 HIE patients when prophylactic barbiturate was used but no reduction in neonatal mortality or long-term outcomes was detected.

The capability of MRI to offer detailed anatomical images of the neonatal brain and evaluate the pattern and severity of perinatal brain injuries acutely has resulted in the more extensive utilization of this neuroimaging modality in newborns with HIE. To improve the prediction of neurodevelopmental outcomes, previous studies have explored and reported on the combination of aEEG within the first 72 h of life and brain MRI at term-equivalent age for preterm infants.^33^ Moreover, qualitative MRI analysis has assisted in identifying structural gray and white matter abnormalities, as well as cerebellar injury, which predict neurodevelopmental outcomes.^34^^,^^35^ Recently, the ability of MRI in term newborns with seizures of mixed etiologies to predicting neuro-disability at 2 years was assessed on the basis of a cohort in the United Kingdom. Similar to the present study, Osmond et al. reported that the risk for later epilepsy was associated with abnormal MRI findings.^36^ In the result of this study, the MRI NICHD score was proved to be an independent risk factor for abnormal neurodevelopment. Moreover, in the recently published volumetric analysis study of infants with HIE, the reduction in brainstem volume coupled with the increase in ventricular volume in infants may serve as a biomarker indicating severe HIE and adverse long-term ND outcomes among infants with HIE who either received Therapeutic Hypothermia (TH) treatment or not.^37^

The main strength of the present study is its unique design, as it analyzed the ability of the combination of aEEG and MRI to predict long-term development. However, this study has several limitations. First, this was a retrospective study, which might be unable to fully confirm the examined relationships. Second, the study group had a relatively small sample size, originating from a single center. Third, the follow-up rate was relatively low, at 59.3 % (80/135).

## Conclusions

In conclusion, the present study revealed that electrographic seizures do not have predictive value for adverse neurodevelopmental outcomes at 12–24 months of age. Although the incidence of neurodevelopmental impairment or death was significantly higher in the clinical seizure group than in the control group, only cord blood pH and abnormal brain MRI findings were consistently associated with long-term neurodevelopmental outcomes according to multivariate analysis. Brain MRI assessment of the location and extent of injury patterns can serve as a valuable predictor of neurodevelopmental outcomes in neonates with HIE. Further studies with well-designed randomized controlled trials may be warranted to explore the predictive value of seizures for severe brain injury in brain MRI and abnormal neurodevelopmental outcomes.

## Conflicts of interest

The authors have no financial relationship with any organization. No honorarium, grant, or other form of payment was received to produce this manuscript. The authors do not have any sources of financial assistance or potential conflicts of interest.
